# Acquired Hemophilia A Diagnosed Based on Gross Hematuria: A Case Report and Literature Review

**DOI:** 10.1155/2024/2760153

**Published:** 2024-08-12

**Authors:** Kenichi Hata, Junichiro Kato, Yusuke Takahashi, Shun Saito, Keigo Sakanaka, Takahiro Kimura

**Affiliations:** ^1^ Department of Urology Atsugi City Hospital, 1-16-36, Mizuhiki, Atsugi City, Kanagawa-ken 243-8588, Japan; ^2^ Department of Nephrology Atsugi City Hospital, 1-16-36, Mizuhiki, Atsugi City, Kanagawa-ken 243-8588, Japan; ^3^ Department of Urology Jikei University School of Medicine, 3-25-8, Nishishinbashi, Minatoku, Tokyo 105-8461, Japan

**Keywords:** acquired hemophilia, acquired hemophilia A, case report and literature review, gross hematuria, hematuria

## Abstract

Acquired hemophilia A (AHA) is an acquired bleeding disorder caused by neutralizing antibodies (inhibitors) against Coagulation Factor VIII (FVIII:C), causing sudden hemorrhagic symptoms (i.e., subcutaneous bleeding, intramuscular bleeding, and hematuria). Herein, this study is aimed at presenting a case of AHA diagnosed based on hematuria and reviewing patients who were diagnosed with AHA due to hematuria. A 67-year-old woman was referred to Atsugi City Hospital with painless gross hematuria that began 4 weeks before presentation. Contrast-enhanced computed tomography (eCT) revealed an approximately 2 cm mass in the right renal pelvis, and the patient's activated partial thromboplastin time (APTT) was elevated (61.4 s). The day after the endoscopic biopsy, the patient was in shock due to a large retroperitoneal hematoma. Although her condition stabilized after intravenous radioembolization, she underwent emergency surgeries several times because of rebleeding within the next 3 weeks. At that time, APTT was more prolonged at 106.4 s, and the FVIII:C level was 2%. Mixing tests showed an upwardly convex curve after 2-h incubation, indicating the presence of an inhibitor. Factor VIII inhibitor titer was ≥5.1 Bethesda unit (BU)/mL. A combined product of Plasma-Derived Factors VIIa and X (pd-FVIIa/FX), as second-line hemostatic therapy, as well as cyclophosphamide (CYP), were administered after Recombinant Activated Factor VIIa (rFVIIa) had been ineffective. Following this, the Factor VIII inhibitor titer was undetectable, FVIII:C levels were restored, and APTT decreased to within the normal range. Gross hematuria was significantly alleviated. However, the patient died of cytomegalovirus and fungal infections due to prolonged immunosuppressive therapy. Although AHA diagnosed based on hematuria may have a better prognosis than others, there have been occasional cases with severe outcomes. APTT, detected upon initial hematological testing in patients with hematuria, may be a potential indicator of an existing AHA.

## 1. Introduction

Acquired hemophilia A (AHA) is an acquired bleeding disorder caused by neutralizing antibodies (inhibitors) against Coagulation Factor VIII (FVIII:C), causing sudden hemorrhagic symptoms (i.e., subcutaneous bleeding, intramuscular bleeding, and hematuria). Severe bleeding is not uncommon in AHA. The mortality rate of AHA ranges from 8% to 44%, which is significantly higher than that of congenital hemophilia [1]. AHA is an autoimmune disease in which autoantibodies against Factor VIII are produced against a background of connective tissue diseases, malignant tumors, and pregnancy. Hemostasis tests show prolonged activated partial thromboplastin time (APTT) and decreased FVIII:C in patients with AHA, often coexisting with inhibitors and FVIII:C. AHA treatments include hemostatic therapy for bleeding and immunosuppressive therapy to eliminate inhibitors, but both are often required simultaneously. However, gross hematuria is a frequent complaint with genitourinary disease, and urologists routinely perform workups when this condition is present. Collins et al. reported that the incidence rate of AHA diagnosed based on hematuria was approximately 4% in a 2-year national surveillance study conducted in the United Kingdom [2]. However, there have been no reports focusing on cases of AHA diagnosed based on hematuria. Herein, we present a case of AHA diagnosed based on hematuria and review the relevant literature. This literature review is aimed at highlighting the demographics, clinical characteristics, diagnostic tools, management, and outcomes of patients with AHA due to hematuria.

## 2. Case Presentation

**Figure 1:**
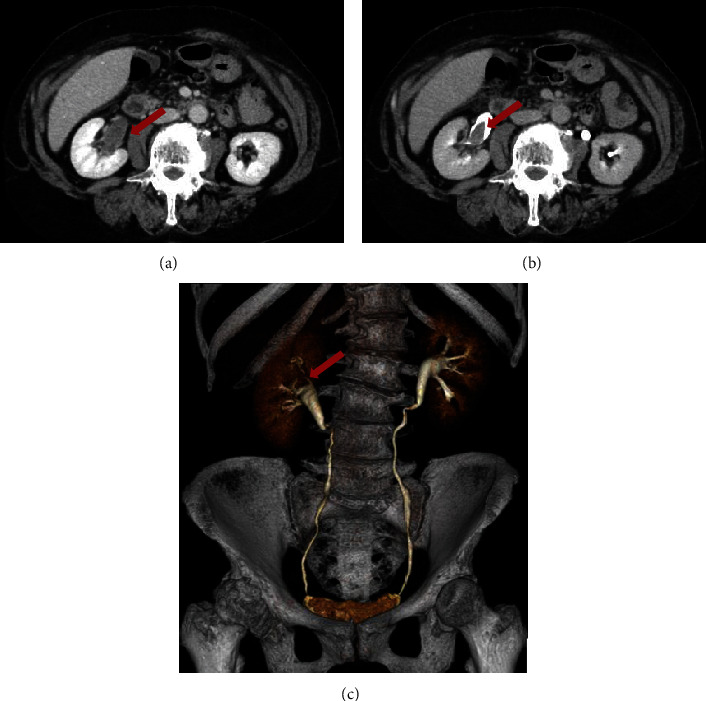
Contrast-enhanced CT reveals an approximately 2 cm mass in the right renal pelvis without evident blood flow. (a) Transverse section of CT delay phase. (b) Transverse section of CT urography. (c) Three-dimensional image of CT urography. CT, computed tomography.

**Figure 2:**
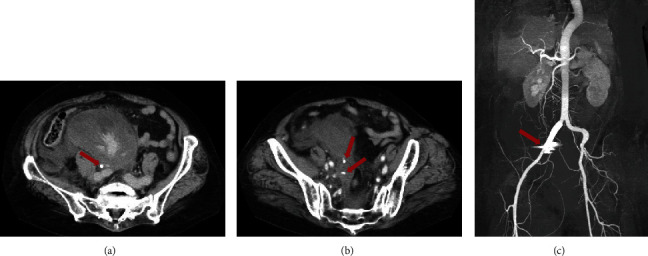
Contrast-enhanced CT reveals a large retroperitoneal hematoma and several bleeding points in the pelvis. (a) Bleeding point in retroperitoneal hematoma. (b) Bleeding points in retroperitoneal hematoma. (c) Bleeding surrounding the right iliac artery. CT, computed tomography.

A 67-year-old woman was referred to Atsugi City Hospital in Japan with painless gross hematuria that began 4 weeks before presentation. She reported a medical history of hypertension and hypothyroidism. She denied having experienced any previous bleeding episodes, even after two previous surgeries (a hysteromyomectomy at age 57 and a right knee replacement at age 64). The patient had no family history of abnormal bleeding and other bleeding manifestations (including the skin) apart from gross hematuria. She had no current or past history of smoking, illicit drug use, alcohol consumption, or medication use that could affect clotting factors. Contrast-enhanced computed tomography (eCT) revealed an approximately 2 cm mass in the right renal pelvis without evident blood flow (Figure 1). Cystoscopy revealed no abnormal findings apart from blood clots. The laboratory findings of the patient are as follows: APTT, 61.4 s (normal range, 25.1–36.5 s); prothrombin time, 12.2 s (range 9.4–12.5 s), 90% (range 70–120%); international normalized ratio, 1.05 (range, 0.9–1.1); hemoglobin, 12.0 g/dL (normal range, 11.6–14.8 g/dL); and platelet count, 185 × 10^9^/L (normal range, 158‐348 × 10^9^/L). The elevated APTT was initially overlooked and not addressed. The infection workup was negative for human immunodeficiency and Hepatitis B and C viruses. Urine cytology revealed the absence of malignant cells. She subsequently underwent endoscopic biopsies of an intermittent bleeding lesion in her right upper calyx to rule out malignancy. This procedure showed an approximately 2 cm blood clot in the right renal pelvis with no histopathological malignancy (histopathological diagnosis and ureteral mucosa). On Postprocedural Day 1, the patient exhibited facial pallor, pale-appearing palpebral conjunctiva, and a reduction in hemoglobin level to 5.1 g/dL. eCT revealed a large retroperitoneal hematoma and several bleeding points in the pelvis (Figure 2). She was placed in the intensive care unit and transfused with red blood cells (16 units) and fresh frozen plasma (8 units) for 3 days. However, gross hematuria persisted, and the retroperitoneal hematoma increased, leading to anemia refractory to aggressive blood transfusion. On Postprocedural Day 3, urgent transcatheter arterial embolization of the right inferior gluteal and internal iliac arteries was performed. Subsequently, the patient's condition temporarily stabilized, and she was discharged to the ward on Postprocedural Day 7.

However, on Postprocedural Day 16, the patient suddenly developed hypovolemic and hemorrhagic shock. Management with mechanical ventilation, norepinephrine, and a more aggressive blood transfusion were required. She was placed in the intensive care unit again. Although we initially placed an endovascular stent graft to block the right internal iliac artery completely, it failed and was subsequently converted to open surgery, where attempts were made to achieve hemostasis using oxidized cellulose, Factor VIII with fibrinogen, and gauze packing. The bleeding had no surgical origin. Consequently, the hematology/nephrology department was consulted to assist in the diagnosis and treatment of the underlying cause of bleeding. Upon hematological workup, the APTT was prolonged at 106.4 s, and the FVIII:C level was 2%. Then, mixing tests showed an upwardly convex curve after 2-h incubation, indicating the presence of an inhibitor. Factor VIII inhibitor Bethesda (Nijmegen) assay showed a titer of ≥5.1 Bethesda unit (BU)/mL. Further testing revealed that the lupus anticoagulant inhibitor, rheumatoid factor, antinuclear antibodies, and extractable nuclear antigens were all negative, and the von Willebrand antigen/function (ristocetin cofactor activity) was elevated. Therefore, the diagnosis of idiopathic AHA was established.

**Figure 3:**
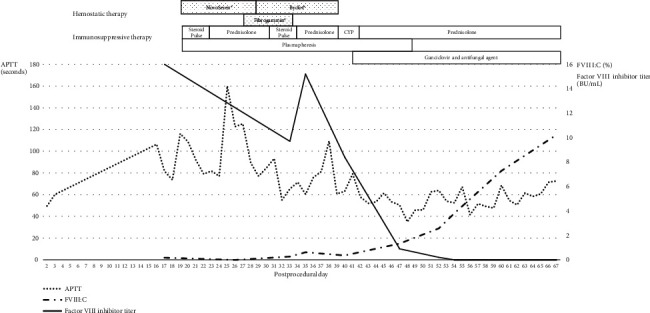
Time course of APTT, FVIII:C, and Factor VIII inhibitor during hemostatic and immunosuppressive therapies. APTT, activated partial thromboplastin; FVII:C, Factor VII activity; BU, Bethesda unit; CYP, cyclophosphamide.

On Postprocedural Day 20, Recombinant Activated Factor VIIa (rFVIIa; NovoSeven®; Novo Nordisk Pharma, Bagsvaerd, Denmark) was administered at a dose of 90 *μ*g/kg every 3 h for 8 days, and immunosuppression was initiated with intravenous methylprednisolone pulse therapy at 1000 mg/day for three consecutive days, followed by 1 mg/kg/day prednisolone. Plasmapheresis was frequently performed, as required. The Factor VIII inhibitor titer remained high, and bleeding persisted. A combined product of Plasma-Derived Factors VIIa and X (pd-FVIIa/FX; Byclot®; KM Biologics Co., Ltd., Kumamoto, Japan) at 120 *μ*g/kg added to highly purified virus-inactivated Plasma-Derived Factor XIII concentrate (Fibrogammin®; CSL Behring, Pennsylvania, United States) was administered on Postprocedural Day 28. On Postprocedural Day 39, cyclophosphamide (CYP) (100 mg daily) was initiated because Factor VIII inhibitors remained high. After this multidisciplinary treatment, a Factor VIII inhibitor titer of up to 15 BU/mL was undetectable, and the FVIII:C levels were gradually restored to 115%. The APTT decreased to within the normal range (Figure 3). Gross hematuria was significantly alleviated. The frequency of blood transfusions and plasmapheresis clinically decreased. She had not received blood transfusions, and her hemoglobin levels had gradually recovered in the last 3 weeks (8.2–9.9 g/dL). However, the patient contracted cytomegalovirus and fungal infections due to immunosuppressive therapy on Postprocedural Day 40. The patient died on Postprocedural Day 72 from cytomegalovirus infection without being discharged from the intensive care unit, although ganciclovir and antifungal agents were prescribed.

## 3. Discussion

AHA is a rare disorder caused by the spontaneous development of autoantibodies against FVIII:C in a nonhemophilic population. A national survey conducted in the United Kingdom reported an incidence rate of 1.48 per million individuals annually, which increased with age until the age of 85 years [2]. However, its incidence may be underestimated because of a lack of knowledge among several nonhematologists [3]. Approximately half the AHA cases are idiopathic. Underlying disorders associated with the occurrence of AHA include malignancy, autoimmune disorders, postpartum infection, and drug-induced factors [2, 4–6]. The frequency of mucosal bleeding in AHA is 31.6%, with the most common site of bleeding being subcutaneous at 53.2% [4]. According to a previous study, of the 149 patients with AHA, only 6 (4.0%) had hematuria [2].

Mingot-Catellano et al. recommended that the presence of AHA should be ruled out in individuals with abnormal bleeding in terms of amount or location, no history of coagulopathy, and unexplained prolonged APTT [3]. Hematuria caused by AHA is a minor symptom, although it is common in urological disorders. However, several urologists may not consider AHA when examining hematuria. Hence, we conducted a literature search on PubMed to investigate the relationship between AHA and hematuria. The search terms were as follows: “acquired hemophilia,” “acquired hemophilia A,” and “hematuria.” Worldwide, 41 AHA cases diagnosed by hematuria, including the present case, were reported in the literature from 1976 to 2023 (Table 1) [1, 7–39]. The ages of patients diagnosed with AHA based on gross hematuria ranged from 24 to 93 years. The age group with the highest incidence was the 60s, accounting for 34.1% of all cases (14/41 cases). Similar to previous reports, AHA is typically found in patients aged >65 years, and its incidence increases with age [2]. This is a feature that distinguishes AHA from congenital hemophilia, where bleeding episodes typically occur in infancy and childhood [40]. The most common bleeding site was subcutaneous in AHA, whereas a characteristic of congenital hemophilia tended to occur deep within the body, such as joints and muscles. The severity of bleeding in hemophilia was related to the measured concentration of the factor (FVIII:C or Factor XI) and was classified as generally predicting bleeding risk [41]. In contrast, FVIII:C and inhibitor titer were not useful for predicting the severity of bleeding events in AHA [3]. In terms of sex, 21 of the reported cases were men (51.2%), and 20 were women (48.8%), which is comparable to previous AHA reports [4, 42]. The bleeding site was specified for 15 patients. Of these cases, nine, three, three, and one were located in the kidney, ureter, bladder, and prostate, respectively. Among them, in one case, each of the ureters and prostates was diagnosed with malignancy. In patients with ureteral cancer who underwent nephroureterectomy, urgent exploration was performed because of the acute progression of anemia and the presence of a large hematoma in the kidney bed postoperatively [21]. In the case of prostate cancer, hematuria was stopped by hormonal therapy, which shrank the prostate tumor [9]. Collins et al. reported that three out of 150 patients had concomitant prostate cancer [2]. The prevalence of underlying malignancies has been previously reported to range from 6.4% to 18.4% [2, 4–6, 43]. Furthermore, the GTH-AH 01/2010 study, a multicenter prospective observational study, reported that underlying malignancy was not a prognostic factor for AHA treatment but an independent predictor of survival [42]. Hence, physicians should consider not only urological cancer but also other malignancies when examining AHA hematuria.

Using univariate analysis, Huang et al. reported that APTT at diagnosis was associated with a shorter relapse of bleeding events. However, it was not associated with multivariate Cox regression analysis [43]. In this study, the median APTT was 72.2 s (range, 41–149.3 s). There was no apparent association between APTT and survival. As mentioned earlier, there was no correlation between FVIII:C, inhibitor titer, APTT, and risk of bleeding in AHA. It might be related to the many types of APTT reagents and the lack of standardization for measuring inhibitor titers. The GTH-AH 01/2010 study reported that complete remission (CR) of AHA was associated with baseline FVIII : C < 1% and World Health Organization (WHO) performance status > 2, whereas survival of AHA was associated with FVIII : C < 1%, WHO performance status > 2, and malignancy [42]. In this study, all 14 cases who had FVIII : C < 1% at diagnosis survived. Two of the 41 patients (4.9%) died. This was lower than those reported in previous studies [2, 42, 43]. This suggests that AHA diagnosed on the basis of hematuria has a good prognosis. Hematuria may be an early symptom of coagulation disorders.

An international expert panel suggested an algorithm for diagnosing AHA. It is recommended that any isolated prolonged APTT should always be investigated for AHA. Heparin, direct thrombin inhibitors, and warfarin should be excluded as the first step. Subsequently, a FVII activity assay and/or APTT mixing study was required [3, 44]. In this case study, during the initial period, we were not aware of AHA, and our facility did not have immediate access to FVII activity assay results. It was the reason prolonged APTT was overlooked. Furthermore, a quantitative test for Factor VIII inhibitor titers was not initially used. It took several days to begin the treatment for AHA. Therefore, in our department, we decided to examine coagulation factors, including FVIII:C and lupus anticoagulant, if APTT is prolonged twice without a history of coagulopathy. If there is an abnormality in coagulation factors, we consult hematologists before the procedure.

The treatments for AHA consist of hemostatic therapy for bleeding and immunosuppressive therapy for CR. rFVIIa, activated prothrombin complex concentrate, and recombinant porcine FVII were recommended options for first-line hemostatic agents [3, 44]. In the EACH2 registry, bleeding control was significantly higher in patients treated with bypassing agents compared with recombinant porcine FVII and DDAVP (93.3% vs. 68.3%; *p* = 0.003) [45]. Furthermore, human FVII replacement therapy is ineffective in the presence of a high Factor VIII inhibitor titer [44]. In our case, rFVIIa was administered as a first-line hemostatic therapy because it was one of the most widely used and effective for severe bleeding [3]. However, severe bleeding persisted despite a sufficient dose and duration. Mingot-Catellano et al. suggested that nonhemostatic causes must be ruled out and the first-line bypassing agent's dose should be increased in subjects without an optimal response [3]. Moreover, the bypassing agent should be switched to the unused one if control was not achieved [3]. There are generally two options, which are rFVIIa and activated prothrombin complex concentrate. In our case, pd-FVIIa/FX, which was available only in Japan, was administered as a second-line hemostatic therapy because rFVIIa had insufficiently improved bleeding tendency and APTT. A preparation called pd-FVIIa/FX, derived from blood donations in Japan, contains FVIIa and FX at a protein weight ratio of 1:10 [46]. We decided to switch to pd-FVIIa/FX as second-line hemostatic therapy because of its longer duration of activity and lower possibility of thrombosis as adverse events [46, 47]. pd-FVIIa/FX may be a promising second-line hemostatic therapy for AHA that is resistant to first-line therapy. The guidelines for immunosuppressive therapy vary according to national experiences [3]. Tiede et al. suggested a simple escalation strategy for eradication of Factor VIII inhibitors, starting with steroid alone (Weeks 1–3), adding CYP (Weeks 4–6), and rituximab (Weeks 7–10), as long as remission was not achieved [42]. On the other hand, in a meta-analysis of immunosuppressive therapy, the CR rate of prednisolone plus CYP combination therapy was higher than prednisolone monotherapy [5]. However, the high CR rate did not coincide with the low mortality rate, and it was thought that a substantial proportion of patients died as a result of complications associated with these agents, mainly neutropenia-related infections, especially in elderly patients [5]. CYP and rituximab are not covered by insurance as AHA immunosuppressive therapy in Japan. In our case, CYP was combined with steroids and plasmapheresis as immunosuppressive therapy after 3 weeks of steroid monotherapy. Following treatment, normalization of APTT, an increase in FVIII:C, and a decrease in the Factor VIII inhibitor titer were observed; however, the patient died from an opportunistic infection caused by prolonged immunotherapy. There are contradictory data regarding the increased risk of infections with steroid regimens in combination or not in the current registries, and there are no clear recommendations for preventing these infections [3]. Archiving the CR of AHA without incurring serious consequences from these infections remains an important challenge in the future.

This study has some limitations. First, it is a literature review of patients with AHA. Regarding the patients with AHA diagnosed with gross hematuria, it is possible that some were unreported and thus not included. Second, pd-FVIIa/FX is available only in Japan. Therefore, there are few reports, and its effects are still unknown. Finally, it was not possible to examine the prognosis of each treatment. Moreover, the AHA treatments in each report, including hemostatic therapy and immunosuppressive therapy, were not standardized.

## 4. Conclusions

Herein, we report a case of AHA that was diagnosed as hematuria and treated with pd-FVIIa/FX as a second-line hemostatic therapy. pd-FVIIa/FX may be a promising second-line hemostatic therapy for AHA that has been resistant to first-line therapy. Although AHA diagnosed based on hematuria may have a better prognosis than others, there have been some cases with severe outcomes. Urologists should recognize that prolonged APTT detected upon initial hematologic testing is a potential indicator of an existing AHA as a screening for hematuria.

## Figures and Tables

**Table 1:** Demographics, clinical characteristics, diagnostic tools, management, and clinical outcomes for patients diagnosed with AHA due to hematuria.

**No.**	**Year**	**Author**	**Nations**	**Age (year)**	**Sex**	**Sites of hematuria**	**Neoplasia**	**Other hemorrhagic features**	**APTT (s)**	**Factor VII activity (%)**	**Factor VII inhibitor (BU)**	**Treatment**	**Outcome**
1	Present	Hata et al.	Japan	67	F	Kidney	None	Retroperitoneal hematoma	61.4	2	>5.1	rFVIIa (NovoSeven®), pd-FVIIa/FX (Byclot®), CYP, steroid, and plasmapheresis	Death
2	1976	Eisenberg and Clark	United States	71	M	Kidney	None		53.9	NA	NA	rFVII and steroid	Alive
3	1980	O'Reilly and Hamilton	United States	62	M	NA	None	Soft tissue hematoma	72	1	250	Diphenylhydantoin was discontinued, Auto-Factor IX (Proplex®)	Death
4	1987	Lottenberg, Kentro, and Kitchens	United States	61	F	NA	None		NA	NA	4	FVII and FIX	Alive
5				81	M	Prostate	Prostate cancer	Hematoma	NA	NA	57	FVII	Alive
6	2000	Lee et al.	Korea	40	F	NA	None		75.7	1.5	27.5	CYP and steroid	Alive
7	2002	Kleinman	United States	62	M	NA	None	Hemarthrosis and ecchimosis	10.5	9	240	rFVIIa (NovoSeven®), FVII, anti-inhibitor coagulation complex (Autoplex®T), CYP, and steroid	Alive
8	2002	Scully, Shublaq, and Oliver	Canada	25	F	NA	None	Subcutaneous hemorrhage and vaginal bleeding	82.5	<0.01	67	rFVIIa (NovoSeven®), CY, steroid, and immunoglobulin	Alive
9	2008	De Pascalis et al.	Italy	24	F	NA	None	Hematoma	80	1.1	10	FFP, rFVIIa (NovoSeven®), and steroid	Alive
10	2010	Otaki et al.	Japan	53	F	Kidney	None	Subcutaneous hemorrhage	53	1.8	19	Carbazochrome sodium sulfonate, tranexamic acid, CY, and steroid	Alive
11	2010	Lak et al.	Iran	58	F	NA	None	Hematoma	73	2	12	NA	Alive
12				62	F	NA	None	Subcutaneous hemorrhage	100	<1	273	NA	Alive
13				65	F	NA	None	Hematoma	69	<1	25	NA	Alive
14				68	M	NA	None	Subcutaneous hemorrhage and hematoma	87	<1	500	NA	Alive
15				60	M	NA	Malignant tumor (primary unclear)	Hemarthrosis	89	4	39	NA	Alive
16				65	M	NA	None	Hematoma	57	<1	125	NA	Alive
17	2012	Nagao, Yamanaka, and Harada	Japan	77	M	NA	None	Hematoma	68	4	47	Steroid	Alive
18	2012	Rezaieyazdi et al.	Iran	37	F	NA	None	Subcutaneous hemorrhage	95	3.9	200	FFP, CYP, and steroid	Alive
19	2013	Sharma et al.	India	65	F	Kidney and ureter	None	Subcutaneous hemorrhage	55.4	<1	NA	rFVIIa (NovoSeven®), FFP, and steroid	Alive
20	2013	Shander et al.	United States	54	F	NA	None	Subcutaneous hemorrhage	41	2	10	rFVIIa (NovoSeven®), CYP, and steroid	Alive
21	2015	Hosier et al.	Canada	82	M	Kidney	None	Hematomas	71	0.02	6.16	rFVIIa (NiaStase®), CYP, and steroid	Alive
22	2015	Berczi et al.	Hungary	54	F	Ureter	Ureteral cancer		64.9	2	NA	APCC, rFVIIa, rFVII, CYP, and steroid	Alive
23	2015	Kannan, Raj Kumar, and Subramanian	India	50	F	Kidney	None		NA	NA	1.6	APCC (FEIBA®) and steroid	Alive
24	2015	Liang et al.	China	71	M	NA	CMML	Ecchimosis	118.6	6.7	7.4	rFVIIa (KOGENATE®) and immunoglobulin	Alive
25	2015	Washino et al.	Japan	60	M	Bladder	None		74.6	NA	NA	rFVII (ADVATE®)	Alive
26	2016	Niwa et al.	Japan	82	M	Kidney	None		149.3	<1	32.7	Steroid	Alive
27	2017	Ben Haim et al.	Israel	75	M	NA	Lung cancer	Intramuscular hematoma	57	<1	20	rFVII and steroid	Alive
28	2017	Khodamoradi, Nazarinia, and Bazdar	Iran	55	F	NA	None	Subcutaneous hemorrhage and hematoma	NA	NA	NA	APCC (FEIBA®), desmopressin, rituximab, and steroid	Alive
29	2018	Burdziak et al.	Poland	50	F	Ureter	None	Retroperitoneal hematoma	NA	7	2	rFVIIa, FVII, CYP, and steroid	Alive
30	2018	Schmidt-Bowman et al.	Lebanon	67	M	Kidney	None		98	NA	384	rFVIIa (NovoSeven®), activated prothrombin complex concentrate (FEIBA®), and rituximab	Alive
31	2018	Yamaguchi et al.	Japan	93	M	NA	None	Subcutaneous hemorrhage and epistaxis	73.1	<1	8.8	rFVIIa (NovoSeven®) and steroid	Alive
32	2019	Yousphi et al.	United States	82	M	Kidney	None		49	Low	Elevated	Rituximab, steroid, and CYP	Alive
33	2020	Hess et al.	United States	91	M	NA	None	Intramuscular hematoma	>100	<1	44	rFVIIa (NovoSeven®), CYP, steroid, and emicizumab	Alive
34	2020	Arain, Muhsen, and Abdelrahim	United States	52	M	NA	Pancreas cancer	Retroperitoneal hematoma	104.9	<1	13.2	rFVIIa, rituximab, and steroid	Alive
35	2020	Mehta and Reddivari	United States	90	M	Bladder	None		48.4	<3	12	rFVIIa, rFVII, steroid, folic acid, and ferrous sulfate	Alive
36	2020	Acik	Turkey	63	M	NA	None		145.5	1	5	Plasmapheresis, CYP, and steroid	Alive
37	2020	Singh, Singh Lubana, and Dabrowski	United States	86	M	NA	None	Oral mucosal bleeding	53.2	<1	1152	APCC, FFP, rituximab, CYP, and steroid	Alive
38	2022	Soliman et al.	Qatar	39	F	NA	None		72.2	2	17.2	None	Alive
39	2023	Kuta et al.	United States	71	F	NA	None	Ecchimosis	145.6	<1	846	FFP and APCC (FEIBA®)	Alive
40	2023	Ryšánková et al.	Czech Republic	60	F	Bladder	Bladder		2.38 (APTT-R)	0.8	33	FFP, rFVIIa (NovoSeven®), CYP, rituximab, and steroid	Alive
41	2023	Arslan Davulcu et al.	Turkey	43	F	NA	None		68	2.2	4.6	Steroid and CYP	Alive

Abbreviations: APCC, activated prothrombin complex concentration; CMML, chronic myelomonocytic leukemia; CY, cyclosporine; CYP, cyclophosphamide; FFP, frozen fresh plasma; NA, not available; rFVII, Recombinant Factor VII; rFVIIa, Recombinant Factor VIIa.

## Data Availability

The authors confirm that the data supporting the findings of this study are available within the article.
